# A Visit to the Electric Shaft

**Published:** 1888-04

**Authors:** 


A VISIT TO THE ELECTRIC SHAFT.—On the 16th ultimo, the editors of this journal visited, in company with a large number of physicians and others, the famous Electric Shaft, in the county of Talliaferro, near the Georgia Railroad, about 65 miles above Augusta.

The proprietors evinced their faith and confidence in the great health restoring properties attributed to this shaft by inviting a large number of medical men, embracing a number of chemists, professors and experts in the medical profession, to examine the locality, to pronounce upon the virtues of the shaft, and say whether or not its healing properties are due to electricity or other causes.

The editors of the *Constitution*, Col. E. P. Howell and Henry W. Grady, were with us as leaders of the excursion train, and well and truly did they do the civiltities of the occasion, making all feel easy, cheerful and comfortable.

On arriving at Hillman, our party was met by a number of carriages and carried to a large, airy and comfortable hotel, situated upon a high hill, overlooking a wide extent of country and presenting a grand and magnificent view.

The hotel and entire property, we learn, is now in charge of a chartered company, known as the "*Georgia Electric Mound Improvement Co.*" The hotel is well constructed and well furnished with all modern conveniences ; the dining room is capacious, the fare good, the proprietors attentive and courteous, sparing no pains to make their guests comfortable.

The surroundings of the Health Resort are certainly fine, and are truthfully described in the following sketch handed us by the proprietors : "The high rolling country, the stately forests, the abundant supply of pure spring water, the absence of swamps or ponds, the red clay soil, the dry and bracing character of the atmosphere, all indicate its freeness from malarial influence, and suggest its great advantages as a resort for pulmonary invalids.

The scenery is as picturesque as is to be seen anywhere outside of a mountainous country, and the walks and drives are as good as can be found in a region like this.

"The historic towns of Washington and Crawfordsville are but a few miles distant. The former was the home of the late Robert Toombs, and is also invested with peculiar interest as being the town in which the Confederate States Cabinet held its last meeting—the house still stands in which that meeting was held. The latter town was the home of the late Alexander H. Stephens, and the house, still standing, in which he lived is an object of interest to all strangers who tarry in its vicinity."

Touching the properties of the shaft, we are not able from so brief an examination to speak positively. We could not detect in our own persons the electric influence which many others claimed to feel.

The "Electric Shaft" is a large excavation about 12 x 50 feet, dug to the depth of about 14 feet in the north side of a large hill. The perpendicular wall on the south side of the shaft is composed of a brownish colored rock containing alum with iron, and perhaps other minerals. In this the electric property is said to exist. The excavation is divided into three compartments, furnished with comfortable seats. Mineral water is brought into these rooms from without by means of pipes. It has a chalybeate taste and is, we doubt not, a valuable water.

While we and others of our crowd could not detect any electric influence, there were some who claimed to feel it perfectly—and there were but few of the ladies who did not feel the influence. It was described by one intelligent lady who had been visiting it daily for some time, and had greatly improved in health, as coming in wave-like impulses.

We have to confess our inability to say whether or not the influence claimed for this shaft is due to electricity or to other causes or forces. That many remarkable cures have resulted at this Health Resort there is abundant evidence in the testimony of many invalids who have found benefit by visiting the shaft and using the water.

It is certainly a good place to pass the time agreeably and pleasantly, and we hesitate not to recommend to any who are in search of recreation or health to to visit the Electric Health Resort.

